# British Columbia Healthy Connections Project process evaluation: a mixed methods protocol to describe the implementation and delivery of the Nurse-Family Partnership in Canada

**DOI:** 10.1186/s12912-015-0097-3

**Published:** 2015-09-17

**Authors:** Susan M. Jack, Debbie Sheehan, Andrea Gonzalez, Harriet L. MacMillan, Nicole Catherine, Charlotte Waddell, For the BCHCP Process Evaluation Research Team

**Affiliations:** School of Nursing, McMaster University, 1280 Main Street West, Hamilton, ON Canada L8S 4K1; Children’s Health Policy Centre, Faculty of Health Sciences, Simon Fraser University, Room 2400, 515 West Hastings Street, Vancouver, BC V6B 5K3 Canada; Department of Psychiatry & Behavioural Neurosciences, McMaster University, Offord Centre for Child Studies, 1280 Main Street West, Hamilton, ON Canada L8S 4K1; Departments of Psychiatry & Behavioural Neurosciences, and of Pediatrics, Offord Centre for Child Studies, McMaster University, 1280 Main Street West, Hamilton, ON L8S 4K1 Canada

**Keywords:** Process evaluation, Home visitation, Public health nurses, Nurse-Family Partnership, Mixed methods, Intervention

## Abstract

**Background:**

The Nurse-Family Partnership is a home visitation program for first-time, socially and economically disadvantaged mothers. The effectiveness of this public health intervention has been well established in the United States; however, whether the same beneficial outcomes will be obtained within the Canadian context is unknown. As part of the British Columbia Healthy Connections Project, which includes a trial comparing Nurse-Family Partnership’s effectiveness with existing services in British Columbia, we are conducting a process evaluation to describe and explain how the intervention is implemented and delivered across five regional Health Authorities.

**Methods:**

A convergent parallel mixed methods research design will be used to address the process evaluation objectives. The principles of interpretive description will guide all sampling, data collection and analytic decisions in the qualitative component of the study. The full population of public health nurses and supervisors (*n* = 71) will discuss their experiences of implementing and delivering the program in interviews (or focus groups). Managers (*n* = 5–15) responsible for this portfolio will also be interviewed annually. Fidelity reports with quantitative data on the reach and the dose of the intervention will be collected and analyzed. Summaries of team meetings and supervisory sessions will be analyzed. Data will be used to compare, corroborate and explain results and variances across the five regional Health Authorities.

**Discussion:**

The process evaluation results will be of immediate instrumental use to the program implementers to inform intervention delivery. Findings will contribute to the emerging body of evidence surrounding: 1) professional nurse home visitation practice issues; 2) best practices for meeting the needs of families living in rural and remote communities; 3) a deeper understanding of how health and social issues such as mental health problems including substance misuse and exposure to intimate partner violence affect a young mother’s capacity to parent; and 4) strategies to support professionals from the primary care, public health and child welfare sectors to work collaboratively to meet the needs of children and families who are at risk or experiencing maltreatment.

## Background

### Nurse-Family Partnership

The Nurse-Family Partnership (NFP) is a home visitation program for first-time, socially and economically disadvantaged pregnant women and mothers with young children. The overarching goals of this evidence-based public health intervention are to improve: 1) pregnancy outcomes; 2) child health and development; and 3) parents’ economic self-sufficiency [1]. Over the past three decades in the United States (US), the effectiveness of the NFP has been tested in three randomized controlled trials (RCTs) with diverse populations of American families living in unique geographic locations: Elmira, New York, Memphis, Tennessee, and Denver, Colorado [2]. Across these trials, consistent and enduring effects of the NFP have been established, including demonstrated improvements in prenatal health behaviours, birth outcomes, and child mental health and developmental outcomes [2]. Findings from the Memphis RCT also conclude that participation in NFP reduces preventable death among nurse-home visited mothers and their children [3]. Furthermore, NFP has been established as the intervention with the best evidence for preventing child maltreatment and other associated outcomes, including reductions in child injuries and emergency room visits [4]. Given the level of evidence attributable to this nurse home visitation intervention in improving the lives of disadvantaged children and families, extensive interest has been expressed by societies outside the US in implementing the NFP. Ongoing evaluation and international NFP research is coordinated by Dr. David Olds (the developer of the NFP) and his team at the Prevention Research Center for Family and Child Health, University of Colorado Denver. In countries outside of the US, there is considerable geographic, demographic, socio-economic, public policy, nursing workforce, and healthcare system variation; it therefore cannot be assumed that the impact of the NFP seen in the US trials will be the same in other countries. Dr. Olds has developed a four-phase model for adapting and evaluating the NFP in international contexts: 1) adaptation: exploration of adaptations needed to deliver the program in local contexts while ensuring fidelity to the 18 core elements [5] of the NFP model; 2) feasibility and acceptability pilot studies: initial small-scale assessment and implementation studies to inform what additional program adaptations may be needed to meet the needs in the new country’s context; 3) RCT: expansion and testing of the intervention in a large-scale RCT to determine NFP’s effectiveness in the specified context; and 4) continued refinement and expansion: if the RCT produces outcomes of public health significance, decisions can then be made regarding wide-scale expansion of the NFP program within the new country [6]. The adaptation of the NFP in England (known there as the Family-Nurse Partnership) is a prime example of how this process for adapting existing evidence-based interventions for new societies can be implemented. In England, the feasibility of implementing this home visitation program along with estimating program costs and determining program impact on key stakeholders was evaluated prior to initiation of the RCT to determine overall effectiveness [7].

### Canadian evaluation of the Nurse-Family Partnership

The first two steps of the process for international NFP replications and evaluation were completed in Hamilton, Ontario, Canada. A concurrent mixed methods pilot study was conducted from 2008 to 2012 to determine overall feasibility and acceptability of the NFP program in Canada with 108 women who met the study eligibility criteria [8, 9]. Based on this work, to successfully deliver NFP in Canada, minor adaptations were made to the US NFP model elements. A list of the model elements to guide subsequent implementations in Canada is provided in Table 1.

**Table 1:** Canadian NFP model elements

Element	Description
1	Client participates voluntarily in the NFP.
2	Client is a first-time mother.
3	Client meets socioeconomic disadvantage criteria at intake.
4	Client is enrolled in the program early in her pregnancy and receives her first home visit no later than the end of week 28 of pregnancy.
5	Client is visited one-to-one, one public health nurse^a^ to one first-time mother or family.
6	Client is visited in her home.
7	Client is visited throughout her pregnancy and the first two years of her child’s life in accordance with the current NFP guidelines.
8	Public health nurses and nurse supervisors are registered professional nurses with a minimum of a baccalaureate degree in nursing.
9	Public health nurses and nurse supervisors complete core educational sessions required by the NFP National Service Office and deliver the intervention with fidelity to the NFP model.
10	Public health nurses, using professional knowledge, judgment, and skill, apply the NFP visit guidelines, individualizing them to the strengths and challenges of each family and apportioning time across defined program domains.
11	Public health nurses apply the theoretical framework that underpins the program, emphasizing self-efficacy, human ecology, and attachment theories, through current clinical methods.
12	A full-time public health nurse carries a caseload of no more than 20^b^ active clients.
13	A full-time nurse supervisor provides supervision to no more than eight public health nurses.
14	Nurse supervisors provide public health nurses clinical supervision with reflection, demonstrate integration of the theories, and facilitate professional development essential to the nurse home visitor role through specific supervisory activities including one-to-one clinical supervision, case conferences, team meetings, and field supervision.
15	Public health nurses and nurse supervisors collect data as specified by the NFP National Service Office (or provincial equivalents) and use NFP reports to guide their practice, assess and guide program implementation, inform clinical supervision, enhance program quality, and demonstrate program fidelity.
16	A NFP implementing agency is located in and operated by a public health agency known in the community for being a successful provider of prevention services to low-income families.
17	A NFP implementing agency convenes a long-term community advisory board that meets at least quarterly to promote a community support system to the program and to promote program quality and sustainability.
18	Adequate support and structure shall be in place to support public health nurses and supervisors to implement the program. Adequate administrative support should also be in place to assure that data are accurately entered into the database in a timely manner.

Adapted from Nurse-Family Partnership [5] ^a^In the Canadian NFP model elements, the term public health nurse replaces the US term of nurse home visitor

^b^Based on results of the Canadian NFP acceptability/feasibility study [8], the standard caseload in Canada was reduced to 20 clients, compared to 25 clients in the US based on differences in annual allowable vacation, length of work-week and client risk levels

Given the findings that the NFP was both feasible to deliver and acceptable to stakeholders in a predominately urban Canadian setting, the McMaster research team sought out provincial partners interested in participating in Phase 3 – the conduct of an RCT to determine overall effectiveness of the NFP to improve maternal and child health outcomes in a Canadian context [10]. As the NFP aligned with key mental health strategic plans and core public health programs, the province of British Columbia (BC) emerged as ready and committed to conducting the RCT. The BC Healthy Connections Project (BCHCP) is the resultant scientific evaluation that includes both an RCT to measure NFP’s effectiveness compared to existing services, and a process evaluation to describe how the NFP is implemented and delivered across the five participating BC Health Authorities (HAs), including describing variances within and between sites [11]. The BCHCP includes one final third project - the Healthy Foundations study that is focused on exploring the biological mechanisms that link intervention and behavioural outcomes in children. This paper outlines the BCHCP process evaluation methods; the BCHCP RCT protocol is the subject of a separate manuscript.

### Process evaluations of complex public health interventions

As a complex intervention, the NFP has multiple interacting components, a range of diverse prenatal, child and maternal outcomes to influence, flexibility in intervention delivery, and sensitivity to local organizational and contextual factors [12]. For example, although PHNs have guidelines on when to conduct home visits, the timing and frequency of home visits will be influenced largely by individual client needs, availability, and engagement in the program. At the level of the individual HAs who are responsible for implementing and delivering the NFP as part of the BCHCP RCT intervention arm and process evaluation, organizational factors such as the level and quality of nursing supervision provided, the time allocated for PHNs to deliver the NFP (e.g., full-time or part-time), and administrative supports and resources for the BCHCP in general have the potential to influence fidelity to the program model. Each HA is also unique in its geography, population characteristics, and the pre-existing culture of partnerships and collaborations that are needed to support and sustain NFP at the community level. This level of complexity creates challenges for evaluating the effectiveness of the intervention and for understanding how the causal mechanisms of the intervention may influence intended outcomes [11].

Increasingly, process evaluations are carried out as part of the overall approach to conducting a comprehensive evaluation of a complex intervention [13]. A process evaluation can help to answer *how* and *why* an intervention succeeds, or fails, within different contexts. The primary functions of the BCHCP process evaluation will be to describe stakeholders’ perceptions and experiences of the intervention components, to assess if the components are delivered with fidelity, to describe planning, implementation and delivery processes and to provide a detailed description of a range of contextual factors that might explain variances across the five HAs. Process evaluations of public health interventions typically include measurements of: fidelity (quality); dose of the intervention delivered (completeness) and received (exposure, including engagement and satisfaction with intervention materials); reach (participation rate); recruitment (enrolment and retention); implementation; and context (individual, organizational, cultural, social, or environmental influences on implementation) [14, 15]. In evaluating a complex intervention, the process evaluation is ideally planned and implemented alongside an RCT. In BC, the NFP will be delivered by 5 unique HAs –our rationale for conducting the process evaluation was so that the different ways in which this intervention is implemented and delivered can be documented and analyzed. This contextual data will be essential for eventually understanding and explaining any potential inter-site variances that emerge from the trial. Data from this process evaluation will also make a substantial contribution internationally related to the adaptations required to successfully deliver NFP to families living in rural or remote communities*.* In addition to enhancing our understanding of the process outcomes related to delivery of the NFP, the data collected within the process evaluation will also enrich our understanding of a wide range of professional nursing practice and education issues.

### Objectives of the BCHCP process evaluation

The overarching objectives for the BCHCP process evaluation are:

1. To determine the extent to which the NFP is delivered with fidelity to the 18 required Canadian model elements (Table 1).
2. To measure the dose of NFP (delivered and received), reach (participation rate throughout pregnancy, infancy and toddlerhood), and recruitment and retention.
3. To explore the acceptability of the NFP intervention to PHNs, supervisors (including the NFP Provincial Coordinator) and public health managers.
4. To describe PHNs’ and supervisors’ experiences of the NFP education program and to identify additional content areas needed for further knowledge and skill development.
5. To explore processes used to support NFP PHNs and supervisors through activities including reflective supervision, coaching and mentoring.
6. To identify contextual factors (individual, organizational, cultural, social and geographic) that influence: organizational adoption and implementation of the NFP; utilization of NFP visit-to-visit guidelines; caseload coordination; location, engagement and retention of NFP clients; sustainability of the NFP in BC; and the convening and sustaining of community advisory boards.
7. To identify adaptations to the NFP model elements to support PHNs and supervisors to meet the needs of clients living in smaller suburban, rural and remote communities.
8. To identify and describe PHNs’ experiences of delivering the NFP to clients and their families exposed to mental health problems including substance misuse, intimate partner violence (IPV), or engagement with the child welfare system.

## Methods

### Public health intervention process evaluation framework

The framework for conducting process evaluations of public health interventions developed by Linnan and Steckler [14] and operationalized by Saunders, Evans, and Joshi [15] was used to guide the planning, development and conduct of this study. Core components of the framework, and how they have been operationalized for the BCHCP process evaluation, are summarized in Table 2.

**Table 2:** BCHCP process evaluation framework

Process evaluation component	BCHCP component operationalization
1. Identify theoretical foundations of the intervention. Construct a logic model to outline intervention components, process and outcomes.	• The theoretical foundations of the NFP are well defined and include theories of human ecology [27], attachment [28] and self-efficacy [29]. The transtheoretical model of behaviour change [30] also informs the work of NFP nurse home visitors, as well as the program logic model [31].
2. Create a theory-informed public health intervention.	• The NFP intervention, systematically developed and evaluated in the US, will be adapted and evaluated for the Canadian context.
3. Create an inventory of process objectives	• For each of the 8 BCHCP process evaluation objectives, a comprehensive list of sub-objectives and topics to be described, measured or explored in the process evaluation, as well as the data sources to be accessed was compiled.
4. Achieve consensus on process evaluation questions to be addressed.	• A multidisciplinary team of researchers with expertise in mixed methods, maternal-child health, professional nursing practice, conducting research with disadvantaged populations and home visiting was established. Building on the principles of integrated knowledge translation, the research team will collaborate and seek ongoing feedback on process evaluation objectives and procedures from the BCHCP Scientific Team, BCHCP Steering Committee, BCHCP Provincial Advisory Committee and BCHCP Regional Evaluation Advisory Committee (as required).
5. Develop quantitative and qualitative data collection tools to address objectives.	• Program fidelity data to be accessed from the BC Ministry of Health.
	• Reporting forms developed to gather data on team meetings and supervisory activities.
	• Interview guides (for 1:1 interviews and focus groups) developed for each phase of the study.
6. Design, implement and conduct rigorous empirical investigation	• The process evaluation will be conducted by adhering to consistent methodological rules and principles to guide both the quantitative and qualitative study components.
7. Collect, manage and clean data	• All qualitative data to be collected by a consistent set of interviews (by the lead principal investigator, and the project Research Coordinator). Interview data to be transcribed verbatim, cleaned and all identifying information removed. Data will be stored, managed and coded in NVivo 10 software.
	• Provincial and HA implementation data to be submitted at least twice a year to the BCHCP Scientific Team.
8. Analyze data	• Content and thematic analysis of the qualitative data will be conducted by designated members of the process evaluation research team.
	• A codebook, with defined codes, will be developed through a process of double-coding and consensus.
	• Quantitative data will be analyzed through the use of descriptive statistics and a series of nested multiple analysis of variance to examine differences between PHNs, within HAs, and across the five HAs.
9. Create user-friendly reports to summarize findings for process objectives.	• Short communication briefs will be developed and disseminated following each phase of data collection (every 6 months) to communicate key findings to the BCHCP Scientific Team, all relevant BC Government and HA policy partners, and to the funder (the Public Health Agency of Canada).
	• This information is one potential source of evidence that the BC Ministry of Health (who holds overall responsibility for NFP implementation in the province) can use to inform HAs about implementation and delivery issues.
10. Refine intervention	• The BCHCP process evaluation data will inform future enhancements and adaptations to the Canadian NFP model which may include specific recommendations for: nurse/supervisor education, IPV interventions, strategies to effectively home visit families in rural and remote communities, and addressing the relationship between primary care, public health and the child welfare sector.

### Design

Within the overarching BCHCP, the process evaluation has been systematically embedded throughout the full conduct of the RCT. A convergent parallel mixed methods [16] research design will be used to address the process evaluation objectives outlined above. This mixed methods design is characterized by the collection and analysis of different –both quantitative and qualitative – but complementary data. Qualitative data from program documents as well as interviews and focus groups with PHNs, supervisors (data from the NFP Provincial Coordinator will be coded and categorized with data from supervisors to ensure anonymity and confidentiality) and senior public health managers will be analyzed to explore and describe how the NFP is implemented and delivered across five diverse regional HAs. Concurrent with this data collection, NFP fidelity reports with quantitative data on the reach and the dose of the NFP intervention will be collected and analyzed. Summaries of the content and number of NFP site team meetings and supervisory sessions will also be provided to the research team for analysis. The reason for collecting both quantitative and qualitative data is to bring together the strengths of both research traditions to compare, corroborate and explain results and variances across the five HAs.

All sampling, data collection and data analysis decisions within the qualitative component of the process evaluation will be guided by the principles of interpretive description [17], an applied qualitative methodology that provides a structure for answering questions that arise within clinical practice [17]. Use of interpretive descriptive methods will enable us to provide a comprehensive description of how the NFP is implemented in BC, and to discover the associations, relationships and patterns among key concepts that are related to the implementation and delivery of this early intervention program, across the HAs. This qualitative approach is one that is used to address issues that have disciplinary relevance (i.e., public health nursing) and to address very specific practice goals. In this study, this approach will thus allow us to expand and extend what is already known about professional practice issues within the disciplinary field of nurse home visitation. Within the process evaluation, we are specifically seeking to understand the practice issue of how the NFP is implemented and delivered within the Canadian context, and more particularly, how this home visiting intervention may be adapted for delivery in smaller suburban, rural and remote areas compared to urban centres.

### Settings

In BC, the Ministry of Health collaborates with the regional HAs that are responsible for the provision of all public health and healthcare services to individuals living within their boundaries. Through the BCHCP, each participating HA is also now responsible for the implementation and delivery of the NFP through their public health services. Four of the five HAs are participating in both the process evaluation and the RCT, while one HA (Northern Health Authority) is only participating in the process evaluation due to its remote geography.

Each HA is further divided into local health areas; the BC Ministry of Health and the participating HAs have identified the particular local health areas that will participate in the BCHCP. In local health areas where it was determined that the conditions were similar to the Hamilton, Ontario context where the pilot study was conducted – namely, that these sites comprise more densely populated large or medium sized urban communities, women who meet the program eligibility criteria will be invited to participate in the RCT. For the purpose of distinction, PHNs delivering the NFP to clients in these local health areas involved in the RCT are considered to be a part of the *expanded* process evaluation.

This distinction was necessary within the four HAs where the RCT is being conducted as there are a number of smaller locations where the BCHCP Scientific Team has determined that it was not feasible to collect RCT data due to either: a) low annual birth rates leading to inadequate sample size; or b) HA preferences. PHNs delivering the NFP in these smaller local health areas are part of the *basic* process evaluation. The fifth HA that is not enrolling any clients in the RCT will also be included in the basic process evaluation.

Some of the HA sites participating in the basic process evaluation are hypothesized to be considerably different compared to the predominantly urban setting where the NFP was first piloted in Hamilton. These HA sites are unique due to: geography (delivery of the program to families living in rural and remote communities); organizational structure (PHNs may not have any other NFP colleagues in the same office; supervisors and PHNs may not be co-located within the same offices); and PHN assignments (they may be allocated to deliver NFP as part of a series of public health responsibilities compared to many of the PHNs in larger sites who will work full-time on the NFP). Thus a significant focus of the *basic* process evaluation will be to determine what adaptations are required to the NFP model of home visitation to meet the needs of families in smaller suburban, rural and remote communities and to identify organizational and professional practice support mechanisms required by the PHNs.

### Sample

**Fig. 1:**
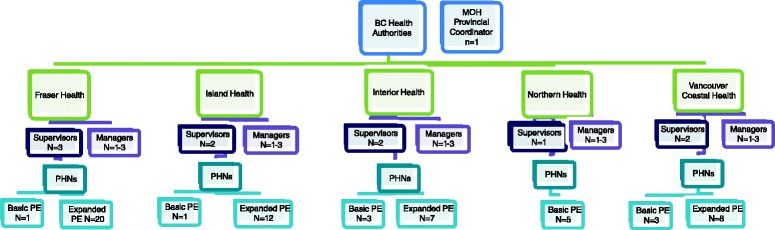
Pursposeful sampling framework for the qualitative arm of the BCHCP process evaluation

Primary data will be collected from all BC NFP PHNs and supervisors (including the NFP Provincial Coordinator). These individuals will be eligible to participate in the process evaluation (basic or expanded) if they: 1) have completed, or are in the process of completing, the NFP education; 2) are delivering the NFP intervention to enrolled participants in the BCHCP (RCT intervention arm or process evaluation); and 3) who speak English. A purposeful sample of senior public health managers from each HA, who have the NFP program within the portfolio of services for which they are responsible, will also be invited to participate. Informed consent will be sought from all NFP service providers eligible to participate in the process evaluation. The total sample size at the commencement of the study is 71 participants including: supervisors + NPF provincial coordinator (*n* = 11) and PHNs (basic process evaluation *n* = 13, expanded process evaluation *n* = 47) (Fig. 1). Through a process of snowball sampling, we estimate inviting 5–15 senior public health managers (1–3 per participating health authority) to also participate. We anticipate that the sample size will increase during the study as new PHNs or supervisors hired to deliver the NFP during the participant recruitment period of the BCHCP will be invited to participate in the process evaluation following the completion of their NFP core education.

### Qualitative data collection

The process evaluation will be characterized by the triangulation of multiple data sources and types to address each research objective. A summary of data sources, types and the frequency of data collection for both the qualitative and quantitative components are summarized in Table 3. Using the principles of interpretive description, qualitative data will be collected from each consenting participant. The NFP PHNs, supervisors and managers will be invited to participate in either an in-depth 1:1 interview (basic process evaluation) or focus groups (expanded process evaluation). Individual interviews will be conducted at regular intervals (approximately every 6 months) until 2018, for a total of eight interviews each. At this rate, with a baseline sample of 11 supervisors (+provincial coordinator), a total of 88 interviews will be conducted with this group, and a total of 104 1:1 interviews with the 13 PHNs (basic process evaluation). However, senior public health managers will be invited to participate in only one annual interview (for a maximum of five interviews per individual; a total of 25–75 interviews across the duration of the study). Multiple interviews are required to document the emerging and ongoing procedures and contextual factors influencing both intermediate and long-term program delivery outcomes. Each participant will be asked to complete a short demographic questionnaire at baseline that will be integrated into the interview facilitation guide and then reviewed for changes during subsequent interviews.

**Table 3:** Process evaluation data sources, types and frequency of collection

Qualitative Data			Quantitative Data	
Data source	Data type	Frequency	Data type	Frequency
PHNs (Basic PE)	1:1 telephone interviews	Every 6 months	• NFP program fidelity data	Quarterly
PHNs (Expanded PE)	• Focus groups (5 per data collection period)	Every 6 months	• Stage of pregnancy (enrolment)	
	• PHNs unable to attend focus group will complete a 1:1 telephone interview		• % of PHN time spent on home visit domains	
			• # home visits	
Supervisors	1:1 telephone interviews	Every 6 months	Supervision records	Completed monthly aggregated every 6 months
Managers	1:1 telephone interviews	Every 12 months		
Field notes	Observations	Ongoing		
Document	Team meeting, case conference summary forms	Completed monthly; aggregated every 6 months		

The content of each interview has been informed by the study objectives – a list of core topics to be explored across the different phases of the study is summarized in Table 4. In-depth 1:1 interviews with supervisors, managers, the NFP provincial coordinator and those PHNs in the basic process evaluation will allow us to collect more nuanced data about adaptations required to deliver the NFP model in a range of geographic contexts. Through the use of interviews, we will be able to explore PHNs’ individual experiences of delivering the NFP and obtain a rich, detailed description of their overall experiences and perceptions with the BCHCP. Each semi-structured 1:1 interview will last approximately 60 min and will be conducted by the Research Coordinator via telephone at a mutually negotiated time. A copy of the interview guide will be shared with each participant prior to the interview, so that the individual can reflect on the questions and develop rich, detailed responses and case examples to share during the interview. Field notes to document observations noted during the interviews, emerging themes and issues, as well as topics to explore in subsequent interviews will be maintained by the interviewer and the research team.

**Table 4:** Summary of primary content – 1:1 interviews & focus groups

Primary Content (1:1 Interviews & Focus Groups)	Public Health Nurses (Expanded & Basic Process Evaluation)	Supervisors (+Provincial Coordinator)	Managers
Interview 1	• Geographic influences (rural, remote, urban) on service delivery	• Role of the NFP Provincial Coordinator	• Role of the Health Authority Manager responsible for the NFP program/BCHCP
	• NFP PHN Education (core, IPV, DANCE, integration)	• NFP planning and implementation phase	• Acceptability of the NFP program
	• Fidelity to model elements	• NFP model elements – acceptability and feasibility	• NFP planning phase
	• Enrolment of women into the PE		• NFP implementation phase
	• NFP acceptability		
Interview 2	• Contextual factors influencing introduction of the NFP into the community	• NFP PHN Education (core, IPV, DANCE, supervisor, integration)	• Role of the Health Authority Manager responsible for the NFP program/BCHCP
	• Client engagement	• Acceptability of the NFP program	• Organizational implementation of NFP
	• Acceptability of the NFP program		
Interview 3	• Supervision	• Implementation and delivery of the NFP	• Role of the Health Authority Manager responsible for the NFP program/BCHCP
	• Intersection between public health & child welfare services	• Supervision	• Organizational implementation of NFP
	• Assessment of ongoing NFP implementation and delivery		
Interview 4	• Clinical practice	• Community collaboration	• Role of the Health Authority Manager responsible for the NFP program/BCHCP
	• Personal and professional impact of home	• NFP community advisory board	• Organizational implementation of NFP
	• visiting vulnerable families		
	• Review of social, geographic and other contextual factors influencing NFP delivery		
Interview 5	• Intimate partner violence	• Supervisor role	• Role of the Health Authority Manager responsible for the NFP program/BCHCP
	• Maintenance of the nurse-client relationship	• BCHCP Implementation	• Organizational implementation of NFP
Interview 6	• Mental health	• NFP staffing	Not applicable
	• Substance use	• Supporting PHNs to work with clients experiencing mental health or substance abuse	
Interview 7	• Public health professional nursing practice issues	• Assessment of ongoing fidelity to NFP model elements	Not applicable
	• Assessment of ongoing NFP delivery		
Interview 8	• Saying good-bye/disengagement process from clients	• Assessment of overall experiences delivering NFP	Not applicable
	• Overall NFP experience		

PHNs enrolled in the expanded process evaluation will similarly be invited to share their experiences and perceptions of the implementation and delivery of the NFP through focus groups conducted within each HA approximately every 6 months. This will provide us with the opportunity to identify unique contextual factors that influence program delivery between and across the four HAs that are also participating in the RCT. Each focus group will include 5–10 participants. Five focus groups will be held in each phase. Over the length of time of the expanded process evaluation, individual PHNs will participate in a total of eight focus groups (overall total number of focus groups for duration of project *n* = 40). The lead researcher who has over 16 years of experience conducting qualitative research (SMJ), or the Research Coordinator (with 8 years of qualitative experience) will facilitate all focus groups, which will be conducted on-site. The lead researcher will be responsible for the main facilitation of the group interviews and the Research Coordinator will manage the logistics and will maintain observational field notes. To minimize the risks of “group think” where participants shape their answers to conform with more dominant or powerful members of the group a range of strategies for posing the questions in different ways to elicit a range of responses will be used in each group [18]. PHNs who are unable to attend the focus group will be invited to complete an abbreviated 1:1 telephone interview.

Concurrent to the focus groups, field site visits will be completed across all five HAs. These field site visits will provide an opportunity to document observations about implementing agencies (i.e., HAs) [14] and provide the researchers with opportunities to develop rapport with study participants in the process evaluation, particularly the NFP supervisors and PHNs. Observations will also be captured in study field notes. Dr. Jack has successfully used this approach in her process evaluation documenting the implementation of the NFP IPV intervention within the context of an RCT to evaluate the effectiveness of the NFP IPV intervention [19] in the US. Initial and subsequent in-person on-site visits to meet key site leaders (e.g., managers, supervisors) and PHNs is anticipated to promote rapid engagement when subsequent data are collected via distance using teleconference, or webinar technology.

Key documents will be collected and analyzed in this process evaluation to corroborate and augment evidence from other data sources and to clarify terms and concepts that might have been mentioned in an interview. A review of documents might identify new issues for exploration in subsequent interviews or focus groups. The following documents will be collected on a quarterly basis by each NFP supervisor: 1) team meeting and case conference summary forms, and 2) a summary of weekly supervision records.

### Quantitative data collection

The BCHCP Scientific Team will be summarizing and analyzing data from NFP program fidelity reports (aggregate data) provided by the BC Ministry of Health. Data contained within these fidelity reports are entered at the local health area level into a health surveillance software application. Data will be reported by implementation location (local health area) and by Health Authority. However, if at any one local unit level, the numbers are small, the Ministry of Health may choose to aggregate the data at the larger HA level only. The following information will be collected and analyzed from the NFP Program Fidelity Reports: 1) stage of pregnancy at time of enrolment; 2) percentage of time spent on each of the six domains during each of the three phases of the NFP program (pregnancy, infancy, toddler); and 3) total number of visits during each of the three program phases. The HAs enter all the data from the NFP nurse assessment forms into the provincial database systems. This information is utilized by the NFP PHNs and supervisors to guide their practice, guide program implementation, inform clinical supervision, enhance program quality, and assess program fidelity. The NFP Program Fidelity Reports are generated automatically from information recorded in the Home Visit/Alternate Encounter form, which is completed after each NFP home visit. The primary purpose of collecting these anonymized and aggregate fidelity data will be to provide a descriptive overview of the fidelity for some of the NFP model elements across local health areas and HAs.

### Data analysis

Qualitative data from the interviews will be analyzed using directed content and thematic analysis, and constant comparative approaches [20]. The analytic process in interpretive description includes broad labeling of concepts through open coding, a synthesis of meanings across codes, the theorizing of relationships through the use of constant comparative methods and the contextualization of data into findings [20]. All interview data will be recorded and transcribed verbatim with any identifying information removed. Transcripts will be reviewed and cleaned by the research team administrative assistant and verified by the Research Coordinator. All textual data (field notes, transcripts, documents) will be imported to NVivo 10.0, a qualitative software package used for overall data management, including coding, searching and indexing.

Both directed (deductive) and conventional (inductive) content analytic procedures will be used to develop the codebook [21]. A set of preliminary codes will be derived from the research questions in the interview guides. However, new codes will be developed and defined as concepts are identified within the data. The codebook will contain the following elements: code name, full definition, instructions on when to apply the code, instructions on when not to apply the code and a brief example of data relevant to that code [22]. The initial code list and code definitions will be circulated to the members of the research team for review. Members of the research team will also independently code a sample of interview data and consistency of code application will be assessed. Based on these findings, the codebook will be reviewed and revised. The Research Coordinator will be subsequently responsible for applying the approved codes to the data and completing all first- and second-level coding.

Quantitative data from NFP Program Fidelity forms and information from the Case Conference forms will be collected and analyzed. Depending on sample size, results will be analyzed and reported at both the local health area and HA levels. The main quantitative analysis will consist of descriptive statistics designed to determine various aspects of program fidelity following recommendations by Boller and colleagues [23]. These fidelity indicators will be evaluated under five broad categories: 1) PHN and supervisor caseloads; 2) duration of the program; 3) service dosage of the program; and 4) content of home visits. According to NFP model fidelity requirements (adapted for Canada) a full-time PHN should carry a caseload of no more than 20 active clients and a full-time supervisor should provide supervision to no more than eight individual PHNs. Measures for this category will include mean monthly PHN and supervisor caseloads and the percentage of PHNs and supervisors at or below required caseload for the observation period. Nurse supervisors provide PHNs with clinical supervision through various activities including one-to-one clinical supervision, case conferences team meetings and field supervision. From the Case Conference forms, number of hours spent in 1:1 supervision (in person or teleconference) per month will be averaged and reported. Time spent in team meetings and conferences will also be averaged and reported. Finally, percentage of time spent on various themes during team meetings will be described.

The NFP seeks to enroll clients early in pregnancy and retain them until the child reaches their second birthday (typically 2.5 years duration). The percent of clients enrolled by 12-, 16-, and after 20 weeks of pregnancy will be described. Duration measures will include: percentage of participants with at least one home visit who remain enrolled at 3, 6, 12, 18 and 24 months; the percentage of participants who do not graduate from the program (at the child’s second birthday); and the mean duration of time spent in the program for those who leave (calculated from the discharge date).

Regular contact with families is one of the key facets of the NFP. NFP guidelines articulate expectations for program dosage varying over the stages of program delivery – pregnancy, infancy and toddlerhood. To assess dosage of the program, we will calculate the percent of participants receiving the intended dosage across all the three stages (90 and 80 % of intended dosage levels) and the number of visits provided between enrollment and date of exit. To fulfill our research objectives, qualitative interview and document data and quantitative NFP Program Fidelity data will be collected in parallel, analyzed separately, and then merged at the interpretation stage.

### Rigor

To ensure the overall quality and trustworthiness of the process evaluation findings, a range of strategies will be applied across all phases of the study to achieve overall data credibility (internal validity), dependability (reliability or the consistency of the findings) and confirmability (neutrality) [24]. A summary of these strategies is provided in Table 5.

**Table 5:** Strategies to achieve rigor across the process evaluation

Criteria	Strategy to achieve rigor	Research phase	Action taken in the process evaluation
Credibility	Time sampling	Data collection	Information will be collected about program delivery and implementation, and how it varies by site, geography and season, across four years of time.
	Engagement in the field	Data collection	Site visits & regular engagement through educational initiatives with PHNs, NFP provincial coordinator, supervisors and managers by researchers. Data will also be collected across a prolonged period of time.
	Reflexivity	For duration of study	Reflexivity will be achieved by the lead researcher and research coordinator maintaining reflexive journals to document and assess the influence of their experiences and perceptions on the qualitative research process.
	Triangulation (data source & type, investigator triangulation)	Data collection & analysis	Data source (PHN, supervisors, coordinator) and type (interviews, focus groups, documents, fidelity data) triangulation will be implemented to cross-check data and to confirm points of convergence or divergence across the dataset. Investigator triangulation will occur as we have created a research team with extensive experience in qualitative research and with a diversity of experiences.
	Member checking	Data collection & analysis	As key themes and issues emerge, they will be discussed and confirmed in interviews/focus groups with subsequent participants.
	Peer examination	Data collection & analysis	This process involves the researchers discussing insights and problems with peers and colleagues. This process will occur in two ways: 1) amongst the members of the BCHCP PE research team and with members of the broader BCHCP scientific evaluation team; and 2) with members of the BCHCP Steering Committee.
	Interviewing process	Data collection	Credibility will be promoted during the interviews and focus groups by reframing questions and participants’ responses, seeking validation of answers, and developing interview guides that are internally consistent [24].
	Researcher credibility	Data collection	The primary interviewers are familiar with the phenomenon under study (home visitation, NFP, program implementation and delivery), have developed strong investigative skills from conducting qualitative research for more than 10 years, and the ability to examine and assess the data from a multidisciplinary perspective [24].
Dependability	Triangulation	Data collection	By collecting multiple types of data (interviews, focus groups, observations recorded in field notes, documents) from multiple sources (PHNs, supervisors, coordinator) dependability of the data is promoted.
	Step-wise replication	Data analysis	Members of the research team will independently code a sample of transcripts for the purpose of early identification of key codes. Researchers will meet to establish consensus around code labels and code definitions.
	Peer examination	Protocol development	As qualitative research is characterized as an emergent design, as decisions are made regarding sampling and data collection, they will be reviewed and discussed in collaboration with the Process Evaluation team of investigators, and as appropriate, with the BCHCP Steering Committee (or appropriate Health Authority partners).
	Dense description of research methods	Sampling, data collection, data analysis	All methodological decisions and actions taken will be documented in the study audit trail.
Confirmability	Maintain audit trail		An audit trail will be maintained by the research coordinator to document all study decisions (and their rationale) and all sampling, data collection and analysis procedures implemented.
	Triangulation	Data collection	As detailed above
	Reflexivity	For duration of study	As detailed above

### Ethical considerations

As the overarching BCHCP is a mixed methods study that includes the RCT and the process evaluation, both studies were submitted as a single application to ten Research Ethics Boards for review. Approval to conduct the BCHCP (inclusive of the process evaluation) was successfully obtained from all ten of these boards – which consisted of five boards from the participating HAs, four boards based in Universities where investigators held their affiliations or faculty appointments, and through the Health Canada and Public Health Agency of Canada Research Ethics Board. The names of the Research Ethics Boards that approved this study are listed in Table 6. Specific to the process evaluation component, informed consent to participate will be sought from all participants. All data will remain confidential and identifying information will be removed from all transcript data. As part of the consent procedure, PHNs and supervisors specifically will be informed about the potential for experiencing distress associated with participation in the process evaluation. The potential to experience distress may occur: 1) when talking to client participants and their families regarding their life circumstances; 2) during reflective supervision; or 3) when discussing their nursing education or role in the BCHCP. Participants will be given the opportunity to take a break or to stop the interview at any point.

**Table 6:** Names of Research Ethics Boards that reviewed and approved BCHCP process evaluation

1. Fraser Health Authority Research Ethics Board, British Columbia
2. Interior Health Authority Research Ethics Board, British Columbia
3. Northern Health Authority Research Ethics Board, British Columbia
4. Vancouver Coastal Health Research Institute Research Ethics Board, British Columbia
5. Island Health Authority Research Ethics Board, British Columbia
6. Hamilton Integrated Research Ethics Board, Ontario
7. Simon Fraser University Research Ethics Board, British Columbia
8. University of British Columbia Research Ethics Board, British Columbia
9. University of Victoria Research Ethics Board, British Columbia
10. Health Canada and Public Health Agency of Canada Research Ethics Board, Ontario

The potential benefits of participating in the process evaluation will also be outlined for participants, including: 1) the experience gained through the NFP education and their involvement in the study is expected to facilitate substantial professional growth and development for participating PHNs and supervisors; and 2) information from this study may be used to help improve programs and services for young, first-time BC (and Canadian) mothers and their children in the future. Findings from the Ontario pilot study [8] suggested that almost all participating PHNs and the local supervisor found the NFP experience to be highly enriching and rewarding. By participating, PHNs and supervisors may be making a lasting contribution to these program improvements.

## Discussion

Historically, much emphasis has been placed on determining and measuring the overall effectiveness of public health interventions through the conduct of RCTs. However, given the complexity of many of these interventions, and the challenges inherent in implementing these programs in different contexts, it is important for researchers to extend their evaluations to include measures of both outcomes and process. In the BCHCP, our process evaluation of the implementation and delivery of the NFP will provide us with important information about fidelity, the quantity of the intervention delivered and received, and the overall quality of the program delivered by HAs. Most importantly, from the process evaluation we will be able to provide a detailed description of the social-political-cultural contexts that are unique to each of the five distinct HAs delivering NFP in BC. This type of data can be useful in interpreting findings from the trial and establishing how generalizable the results will be to other contexts [25]. In BCHCP sites participating in the basic process evaluation (i.e., not enrolling eligible women into the RCT), data from the process evaluation will be essential in providing insights about how PHNs and supervisors were required to adapt model elements and program content to meet the needs of their clients, often residing in smaller suburban, rural and remote areas. This information will be valuable in informing future adaptations to the NFP model for similar populations in Canada and in other international contexts.

This process evaluation has several key methodologic strengths. First, as a mixed methods study, the combination of quantitative and qualitative methods will result in findings that offer a more comprehensive understanding of how the NFP is implemented in BC; the integration of the contextual qualitative data will ensure that the findings can be utilized conceptually by health decision makers. It has been suggested that research that includes three key domains (process, content, and outcomes) will be more likely to result in policy change or evidence-informed decision making within the public health arena [26]. The BCHCP, including findings from both the RCT and the process evaluation described here, therefore has a high likelihood of influencing policy change, given its mixed methods approach addresses all three domains. Second, the triangulation of multiple data types from a range of sources (including PHNs, supervisors and managers) across the full geographic region where the NFP is being evaluated will provide results with a high level of credibility and trustworthiness. With information from the process evaluation, we will also be able to explain variances in implementation and outcomes (measured in the RCT) across and within the HAs. Another significant strength of the process evaluation, and the overall BCHCP, is the strong, collaborative partnership that has been developed between the BCHCP Scientific Team and the senior policy-makers within BC’s Ministries of Health and Child and Family Development. As with all aspects of the BCHCP to date, relevant BC Government and HA decision-makers have been and will continue to be engaged in providing feedback on the process evaluation sampling and data collection procedures and tools. As we move into the BCHCP RCT and process evaluation, there will continue to be ongoing dialogue and problem-solving using our established research-policy partnership governance structures. These existing relationships and structures thus result in opportunities to ensure that important information about implementation, model fidelity and program delivery is disseminated in a timely fashion to policy-makers, informing their decisions.

One limitation of the process evaluation is the lack of opportunity to collect qualitative data from the pregnant women and first-time mothers, as well as their partners and family members, who are enrolled in the BCHCP and receiving NFP. While data about the dose of the intervention received will be captured at an aggregate level, we will not be able to collect information about their experiences of engaging in the NFP home visitation program and their perceptions on how this intervention influences their capacity to parent.

Overall, the results of this process evaluation will be of immediate instrumental use to the policy-makers to inform ongoing implementation and delivery of the NFP. Beyond the context of BC, the findings from our study will contribute to the emerging body of evidence surrounding: 1) professional practice issues in the field of nurse home visitation, 2) best practices for adapting the NFP model elements to meet the needs of families living in smaller suburban, rural and remote communities; 3) a deeper understanding of how social and health issues such as mental health problems including substance misuse and IPV impact a young mother’s capacity to parent; and 4) strategies to support professionals from the primary care, child welfare and public health sectors to work collaboratively to meet the needs of children and families at risk or experiencing maltreatment. If the BCHCP RCT demonstrates the effectiveness of the NFP compared with existing services, for the first time in the Canadian context, findings from the process evaluation will be invaluable in informing wide-scale implementation efforts in Canada. For example, process evaluation findings related to PHNs’ and supervisors’ experiences of completing the NFP education components would inform the development of a Canadian-specific NFP model of education delivery. Valuable information pertaining to strategies for the recruitment and retention of both PHNs and clients will also be available. Finally, the findings will be of great interest and assistance with evaluating and implementing the NFP internationally.

## Abbreviations

BC: British Columbia
BCHCP: British Columbia Healthy Connections Project
HA: Health authority
IPV: Intimate partner violence
NFP: Nurse-Family Partnership
PHN: Public health nurse
RCT: Randomized controlled trial
US: United States

## References

[1] Nurse-Family Partnership; http://www.nursefamilypartnership.org (2011). Accessed 9 May 2015.

[2] Olds DL, Sadler L, Kitzman H (2007). Programs for parents of infants and toddlers: Recent evidence from randomized trials. J Child Psychol Psy.

[3] Olds DL, Kitzman H, Knudtson MS, Anson E, Smith JA, Cole R (2014). Effect of home visiting by nurses on maternal and child mortality: results of a 2-decade follow-up of a randomized clinical trial. JAMA Pediatrics.

[4] MacMillan HL, Wathen CN, Barlow J (2009). Interventions to prevent child maltreatment and associated impairment. Lancet.

[5] Nurse-Family Partnership: Nurse-Family Partnership model elements. http://www.nursefamilypartnership.org/communities/model-elements (2011). Accessed 9 May 2015.

[6] Prevention Research Center for Family and Child Health: Nurse-Family Partnership international program. http://www.ucdenver.edu/academics/colleges/medicalschool/departments/pediatrics/research/programs/prc/research/international/Pages/international.aspx (2015). Accessed 9 May 2015.

[7] Barnes J (2010). From evidence-base to practice: implementation of the Nurse Family Partnership programme in England. J Child Serv.

[8] Jack SM, Busser LD, Sheehan D, Gonzalez A, Zwygers EJ, MacMillan HL (2012). Adaptation and implementation of the Nurse-Family Partnership in Canada. Can J Public Health.

[9] Kurtz Landy C, Jack SM, Wahoush O, Sheehan D, MacMillan L and NFP Hamilton Research Team (2012). Mothers experiences in the Nurse-Family Partnership program: a qualitative case study. BMC Nurs.

[10] Jack SM, MacMillan HL. Adaptation and evaluation of the Nurse-Family Partnership in Canada. Early Childhood Matters 2014, June: http://www.bernardvanleer.org/Responsive-parenting-a-strategy-to-prevent-violence. Accessed 9 May 2015.

[11] Children’s Health Policy Centre: BC Healthy Connections Project. http://childhealthpolicy.ca/bc-healthy-connections-project/ (2015). Accessed 9 May 2015.

[12] Medical Research Council (2008). Developing and evaluating complex interventions: new guidance.

[13] Lewin S, Glenton C, Oxman AD (2009). Use of qualitative methods alongside randomised controlled trials of complex healthcare interventions: methodological study. BMJ.

[14] Linnan L, Steckler A, Steckler A, Linnan L (2002). Process evaluation for public health interventions ad research: an overview. Process Evaluation for Public Health Interventions and Research.

[15] Saunders RP, Evans MH, Joshi P (2005). Developing a process-evaluation plan for assessing health promotion program implementation: a how-to guide. Health Promot Pract.

[16] Creswell JW, Plano Clark VL (2011). Designing and Conducting Mixed Methods Research.

[17] Thorne S (2008). Interpretive Description.

[18] MacDougall C, Baum F (1997). The devil’s advocate: a strategy to avoid groupthink and stimulate discussion in focus groups. Qual Health Res.

[19] Jack SM, Ford-Gilboe M, Wathen CN, Davidov DD, McNaughton DB, Coben JH, Olds DL, MacMillan HL, NFP IPV Research Team (2012). Development of a nurse home visitation intervention for intimate partner violence. BMC Health Services Res.

[20] Thorne S, Reimer Kirkham S, O’Flynn-Magee K (2004). The analytic challenge in interpretive description. Int J Qual Methods.

[21] Hsieh H, Shannon SE (2005). Three approaches to qualitative content analysis. Qual Health Res.

[22] MacQueen KM, McLellan E, Kay K, Milstein B (1998). Codebook development for team-based qualitative analysis. Cultural Anthropol Methods.

[23] Boller K, Daro D, Del Grosso P, Cole R, Paulsell D, Hart B (2014). Making replication work: building infrastructure to implement, scale-up, and sustain evidence-based early childhood home visiting programs with fidelity.

[24] Krefting L (1991). Rigor in qualitative research: the assessment of trustworthiness. Am J of Occup Ther.

[25] Wells M, Williams B, Treweek S, Coyle J, Taylor J (2012). Intervention description is not enough: evidence from an in-depth multiple case study on the untold role and impact of context in randomised controlled trials of seven complex interventions. Trials.

[26] Brownson RC, Chriqui JF, Stamatakis KA (2009). Understanding evidence-based public health policy. Am J Public Health.

[27] Bronfenbrenner U (1979). The Ecology of Human Development: Experiments By Nature and Design.

[28] Bowlby J (1969). Attachment and Loss.

[29] Bandura A (1977). Self-efficacy: toward a unifying theory of behavioral change. Psych Rev.

[30] Prochaska JO, DiClemente CC, Miller WR, Heather N (1986). Toward a comprehensive model of change. Treating Addictive Behaviors: Processes of Change.

[31] O’Brien R. Nurse-Family Partnership’s theory of change logic model http://www.nursefamilypartnership.org/assets/PDF/Communities/TOC-Logic-Model (2008). Accessed 9 May 2015.

